# How to make an aortic root replacement simulator at home

**DOI:** 10.1186/s13019-015-0223-z

**Published:** 2015-02-06

**Authors:** Kasra Shaikhrezai, Maziar Khorsandi, Edward T Brackenbury, Sai Prasad, Vipin Zamvar, John Butler, Geoffrey Berg

**Affiliations:** 1 Department of Cardiac Surgery, Golden Jubilee National Hospital, Glasgow, UK; 2 Department of Cardiac Surgery, Royal Infirmary of Edinburgh, Edinburgh, UK

**Keywords:** Education, Aortic root replacement, Aortic valve

## Abstract

There is a paucity of low-fidelity and cost-efficient simulators for training cardiac surgeons in the aspects of aortic root/valve replacement. In this study we addressed this training challenge by creating a low-fidelity, low-cost but, at the same time, anatomically realistic aortic root replacement simulator for training purposes. We used readily available, low cost materials such as lint roller tubes, foam sheet, press-and-seal bags, glue, plywood sheet, heat-shrink sleeving tubes and condoms as the basic material to create a low-fidelity, aortic root, training simulator. We constructed a multi-purpose, anatomically realistic aortic root simulator using the above materials, both time- and cost-efficiently, using the minimum of surgical equipment. This simulator is easy to construct and enables self-training in major techniques of aortic root replacement as well as in stentless valve implantation for trainees in cardiac surgery.

## Introduction

It is recognised that simulation plays an important rôle in training surgeons and is a component of residency training programmes [1]. Due to the complexity of cardiac surgery, simulation technology in this field is not well-developed and the cost of most simulators remains a major obstacle for everyday use [2].

Aortic root surgery is technically demanding. One of the challenges of training residents to perform aortic root surgery is matching the trainee skills with case complexity [3]. Trainees may not obtain adequate operator experience and exposure to aortic root disease during a cardiac surgery training programme, simulation could offer a solution to mitigate this. In this study, we aimed to address this training challenge by creating a low-fidelity, low-cost but anatomically realistic, aortic root replacement simulator for training purposes.

## Review

### Materials and methods

**Figure 1:**
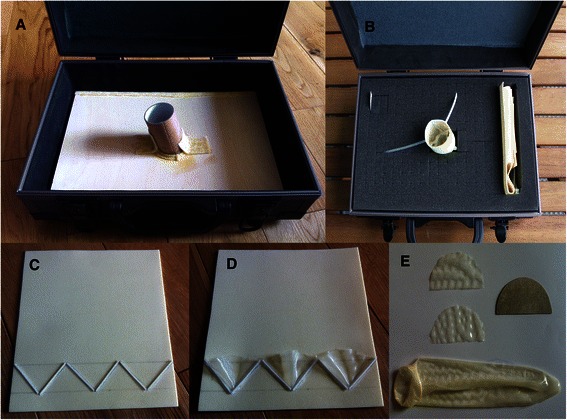
**How to construct the low fidelity aortic root simulator. A)** The simulator from surgeon’s view; **B)** The simulator skeleton: flight briefcase, lint roller tube, plywood sheet; **C)** Press-and-seal segments attached to the foam sheet to create the annulus and the commissures **D)** Semi-circles cut out from condom in cusps position **E)** Cusps cut out of condom.

**Figure 2:**
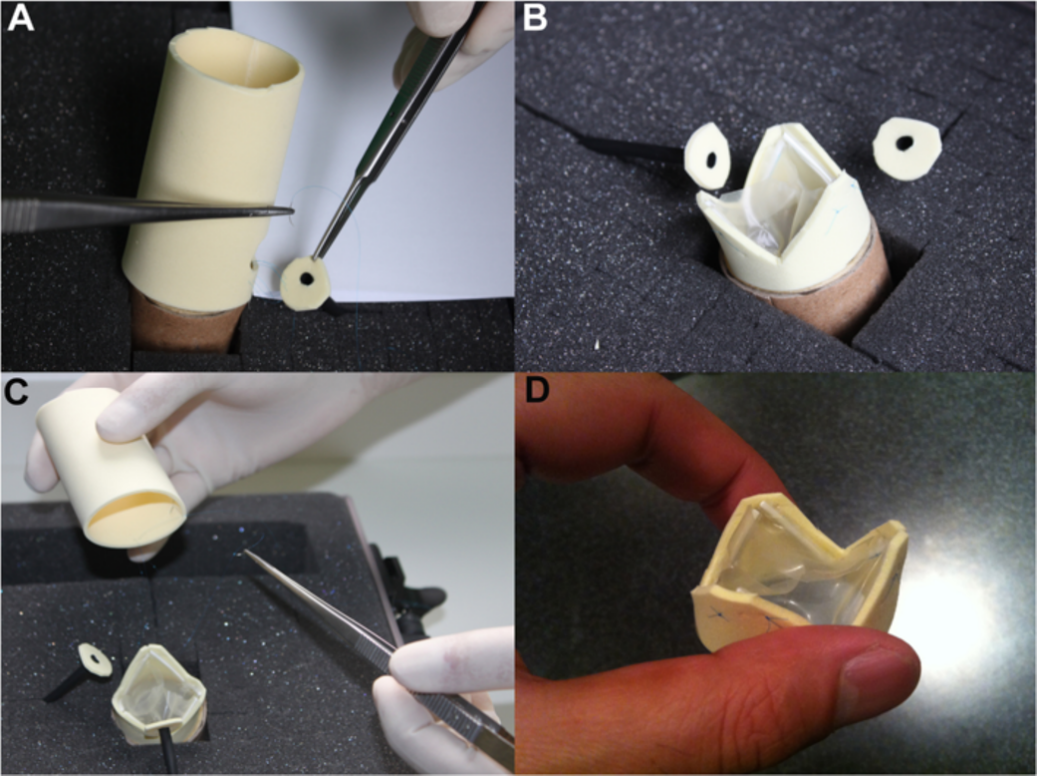
**The training utilities of the low fidelity aortic root simulator. A)** Coronary button re-implantation following implantation of a valved conduit in the root position **B)** Mobilised coronary buttons and the spared aortic valve for valve-sparing aortic root surgery **C)** Valve-sparing aortic root replacement **D)** Stentless aortic valve.

A small flight briefcase (N = 1, $ 50.20, reusable) is the simulator casing. It can be carried easily and, when opened, is arranged from the surgeon’s viewpoint as in the operating theatre (Figure 1A). An oblique lint roller tube (N = 1, $ 0.60) is secured on a plywood sheet (N = 1, $ 9.70, reusable) as the simulator skeleton (Figure 1B). The tube is in a sponge working field. The root and ascending aorta are replicated by a rolled and glued foam sheet with two heat-shrink sleeving tubes (N = 2, $ 0.33, non-reusable), replicating the coronary arteries, attached to it (Figure 2). The aortic valve and the commissures are reproduced by a condom (N = 1, $ 0.70, non-reusable) and a ‘press-and-seal’ sandwich bag plastic sealer (N = 60, $ 2.6, reusable) respectively. The pattern of aortic root and the measurements are mapped on the foam sheet (N = 1, $ 0.83, non-reusable). For example, to design a 4 cm aortic root simulator the following measurements need to be taken into account: Foam sheet width: 13.6 cm, Profile height (distance between the upper and lower lines): 2.2 cm, Divide upper line into three segments of 4.5 cm, Divide the lower line into two segments of 4.5 cm in the middle and one segment of 2.25 cm on either side of the middle segments, six length of 3.2 cm press-and-seal to form the annulus in zigzag pattern connecting the upper and lower line by the segments.

The press-and-seal strip is cut out into multiple segments which are sewn or glued by super glue (N = 1, $ 2.6, re-usable) to the foam sheet (Figure 2). The aortic valve cusps are cut out of the condom in a semi-circular fashion, which is then held firmly by the plastic sealer. The semi-circle cusps need to be cut out with a diameter of 1.5 – 2 times larger than the distance between the commissures so when they are held by the press-and-seal segments can be easily trimmed to mimic a well-coapting valve. A strip of plain felt sheet (N = 1, $ 0.83, reusable) is attached around the lint roller tube as the sewing cuff (annulus) for implantation of a valved conduit or aortic valve.

### Results/Functions

We were able to construct a multipurpose, anatomically realistic aortic root replacement simulator using the above materials both time and cost efficiently, using minimal surgical equipment. The simulator enables the residents to learn and practice procedures including; Coronary buttons re-implantation (Figure 2A), Aortic root replacement, Valve-sparing aortic root replacement (Figure 2B,C) and Stentless aortic valve replacement (Figure 2D). The foam sheet forms the core of the simulator, which allows the trainees to experience a perfectly simulated tissue handling and accurate angle of needle attack in complex aortic root and stentless valve surgery.

## Discussion

Clinical medicine is increasingly concerned with patient safety and a consultant-delivered, better quality health care possibly at the expense of bedside teaching and practice for residents and registrars [4]. Hence, in the recent years medical education has seen a major change in the medical education paradigm with a shift towards using medical simulation in training [4]. Evidence shows that simulation in training surgical residents and registrars leads to an improvement in technical performance in the operating theatre [4]. However, despite the technical advantages it confers on training, simulation can be extremely costly for trainee surgeons [5].

The foundation of the design of our simulator is of “low-fidelity” properties, which would allow its reproduction in an easy and inexpensive manner. Hence the simulation is not an exact replica of the aortic root anatomy such as annulus topography. We therefore recommend that users familiarise themselves with the real aortic root anatomy prior to practicing with the simulator. As the simulator is designed to be used in a dry lab setting exclusively, the anastomosis and root construction quality control can not be achieved by liquids. The integrity of the anastomosis, we believe, can be assessed by its aesthetic appearance. It is possible to test the strength of the anastomosis if the trainer/trainee wishes, with an instrument e.g. one arm of the Gerald/De Bakey forceps or a nerve hook if this is available, could be gently slid through the anastomosis and carefully tugged on.

## Conclusion

In conclusion, our design provides an efficient construction of a low-fidelity, aortic root simulator and enables self-training in techniques of aortic root replacement and stentless valve implantation for the cardiac surgeons.
